# Surgical Excision and Skin Grafting for Severe Chronic Scalp Pyoderma: A Case Report and Literature Review

**DOI:** 10.7759/cureus.87162

**Published:** 2025-07-02

**Authors:** Ayane Ezaki, Suzuki Yushi, Mayuko Okura, Masahiro Toriumi, Junji Takano

**Affiliations:** 1 Plastic and Reconstructive Surgery, Japan Community Healthcare Organization (JCHO) Saitama Medical Center, Saitama, JPN; 2 Plastic and Reconstructive Surgery, Keio University School of Medicine, Tokyo, JPN

**Keywords:** acne keloidalis nuchae, dissecting cellulitis of the scalp, skin grafting, suppurative scalp disease, surgical excision

## Abstract

Chronic suppurative diseases of the scalp, including acne keloidalis nuchae (AKN), dissecting cellulitis of the scalp (DCS), and folliculitis decalvans, are characterized by the presence of persistent abscesses, fistulas, and progressive scarring. The treatment of these conditions often presents a challenge, requiring both pharmacological and surgical interventions. We present herein two cases of long-standing, chronic, refractory suppurative scalp conditions that were successfully treated with surgical excision and subsequent skin grafting. The first case is that of a 53-year-old man with AKN, characterized by extensive abscesses and scarring in the occipital region. The second case is that of a 61-year-old man with DCS, exhibiting recurrent folliculitis and abscesses. Both patients underwent a complete excision of the affected areas followed by full- or split-thickness skin grafts, resulting in favorable cosmetic outcomes. Histopathological examination in each case confirmed the diagnoses of AKN and DCS. These case reports underscore the effectiveness of surgical intervention in managing resistant cases of severe chronic suppurative diseases of the scalp, with good postoperative outcomes. This finding highlights the potential for improved quality of life with timely surgical management among patients suffering from these diseases.

## Introduction

Chronic suppurative diseases of the scalp are characterized by the formation of chronic abscesses and fistulas in the scalp, as well as repeated cycles of inflammation and scarring. Depending on its clinical presentation, this condition has various names, including acne keloidalis nuchae (AKN), dissecting cellulitis of the scalp (DCS), and folliculitis decalvans (FD); however, it is thought that these conditions share the same underlying pathology. These diseases involve progressive inflammation associated with follicular obstruction and destruction, often making treatment challenging. While pharmacological treatments such as antibiotics and immunosuppressants have been reported as therapeutic options, surgical intervention can yield cosmetically acceptable and favorable outcomes. We report herein two cases involving long-standing abscess formation and refractory scarring that were successfully treated with surgical intervention, resulting in favorable postoperative outcomes. A review of the relevant literature is also included in this discussion.

## Case presentation

Case 1

**Figure 1:**
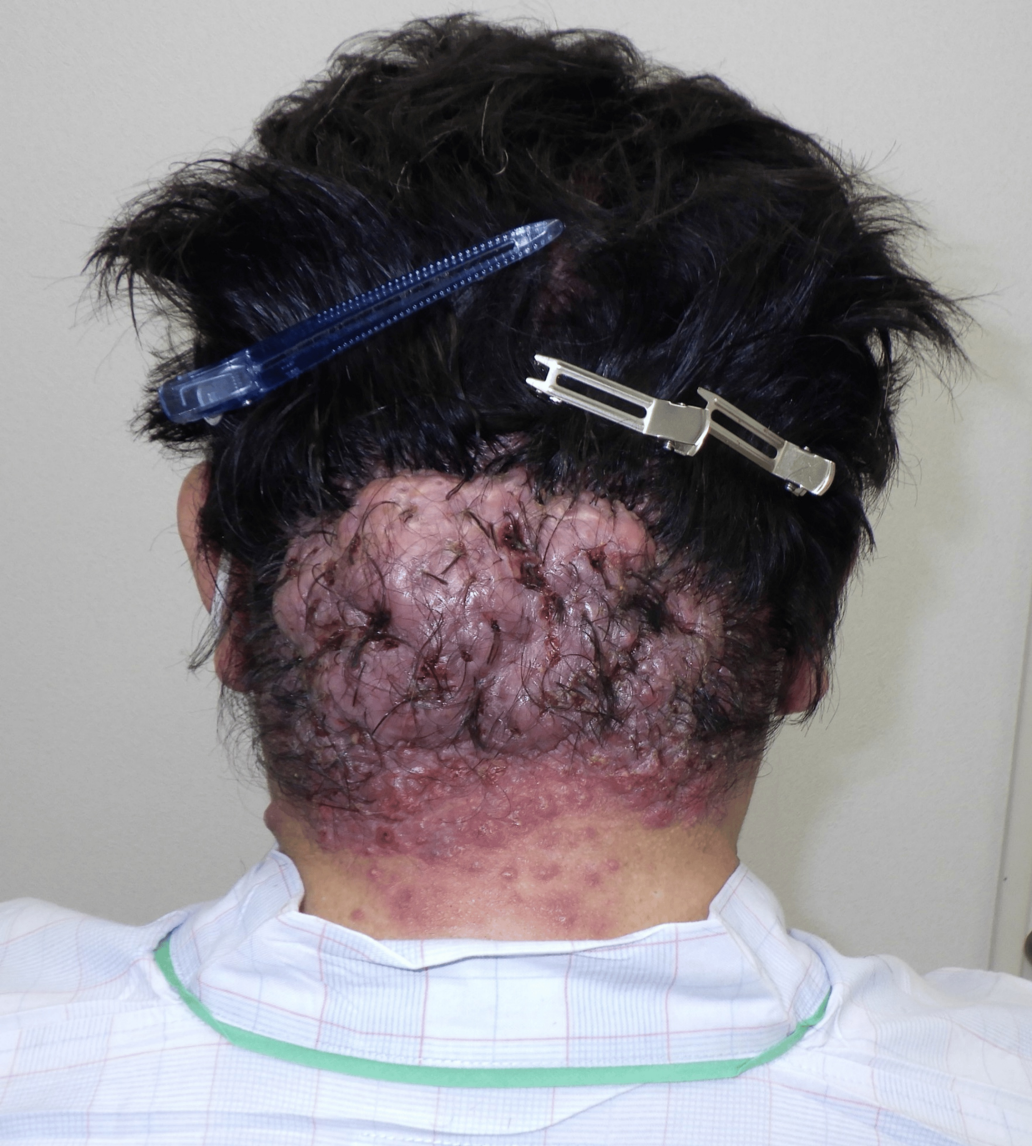
Preoperative photo A 15 × 10 cm mass observed in the occipital region of the scalp

A 53-year-old man presented with a mass on the occipital region of his scalp. As a construction worker, he routinely wore a helmet for extended time periods. The patient reported that he had been aware of the mass for at least 10 years; however, as his symptoms had worsened over the past year, he was encouraged to visit our hospital. Although his medical history included hypertension, diabetes mellitus, and dyslipidemia, he had not been receiving treatment for these conditions. A physical examination revealed a protruding 15 × 10 cm lesion in the occipital region of his scalp. The central area of the lesion displayed hair loss, with some regions discharging pus (Figure 1).

**Figure 2:**
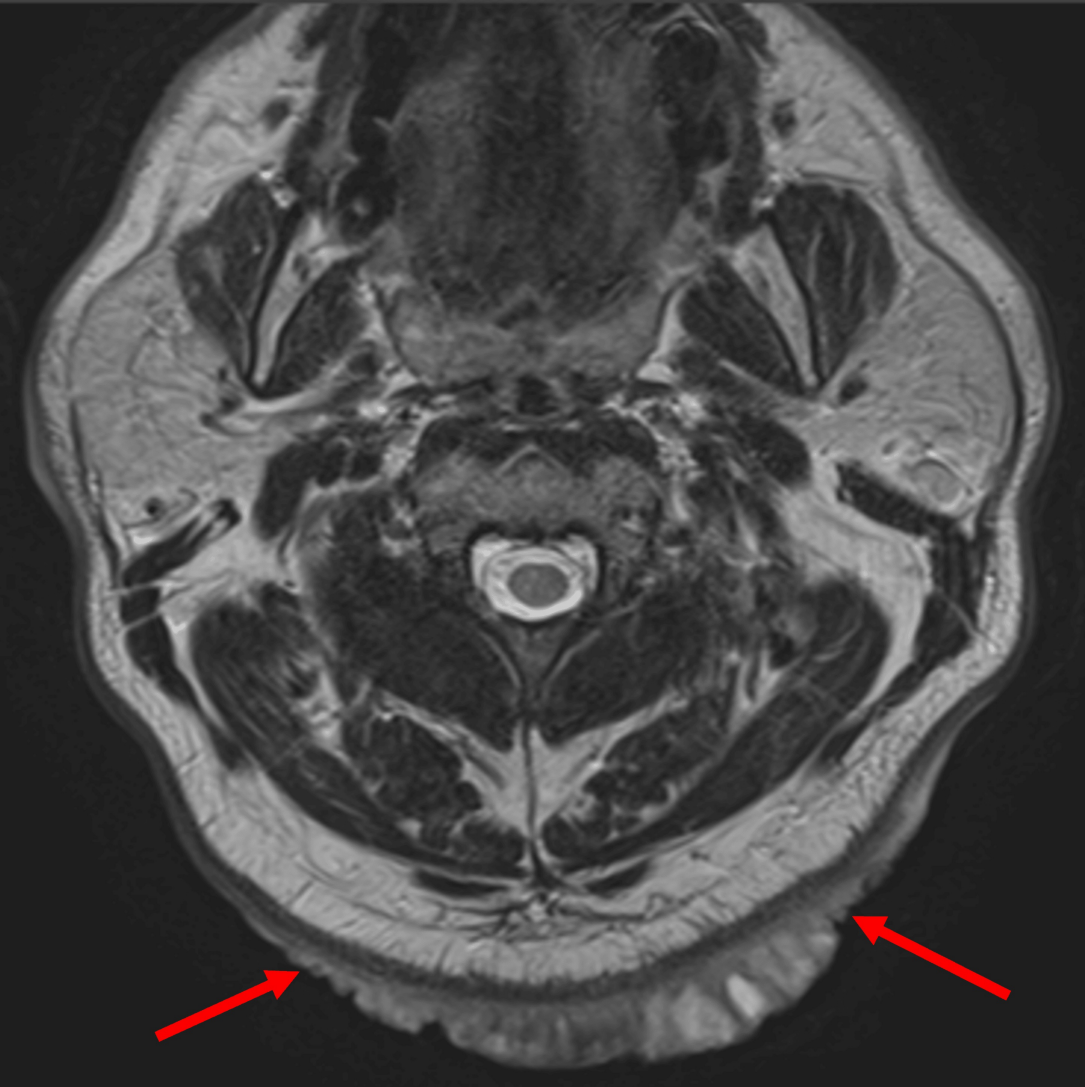
Preoperative MRI T2-weighted magnetic resonance imaging (MRI) showing a high-signal area extensively spreading within the subcutaneous tissue from the occipital region of the scalp to the posterior neck. Infiltration into the subcutaneous fat layer was not observed Red arrow: tumor

**Figure 3:**
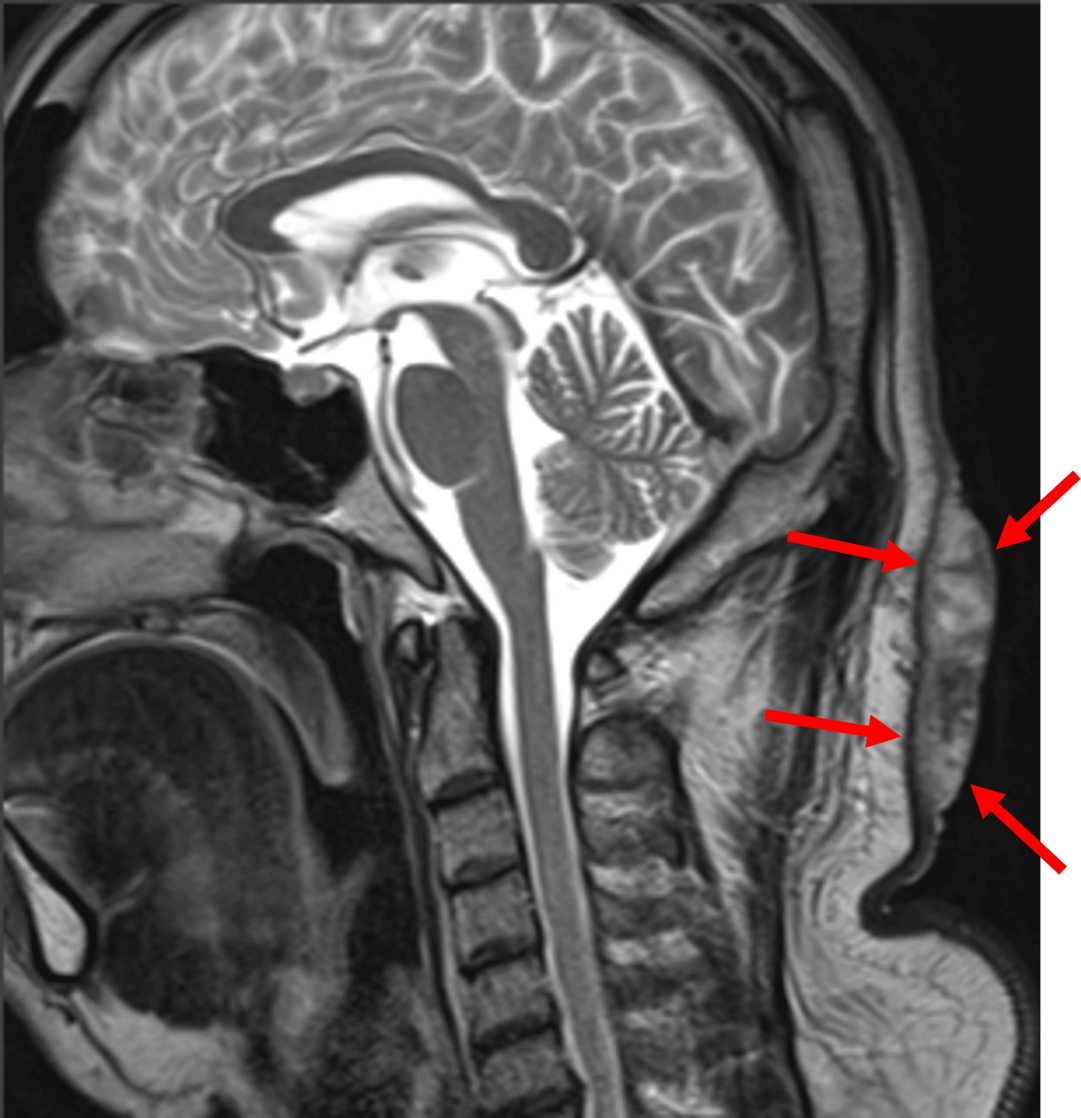
Preoperative magnetic resonance imaging (MRI) Sagittal view of the MRI

Laboratory tests showed leukocytosis with a white blood cell count of 10,400/µL, random blood glucose of 297 mg/dL, National Glycohemoglobin Standardization Program hemoglobin A1c of 9.2%, and C-reactive protein of 3.24 mg/dL, indicating poor glycemic control and mild inflammation. T2-weighted magnetic resonance imaging (MRI) revealed a wide high-signal area extending throughout the subcutaneous tissue from the occipital to posterior cervical regions, although it did not infiltrate the subcutaneous fat layer. Due to the extent of the lesion, surgical excision was determined as the best treatment course (Figures 2, 3).

**Figure 4:**
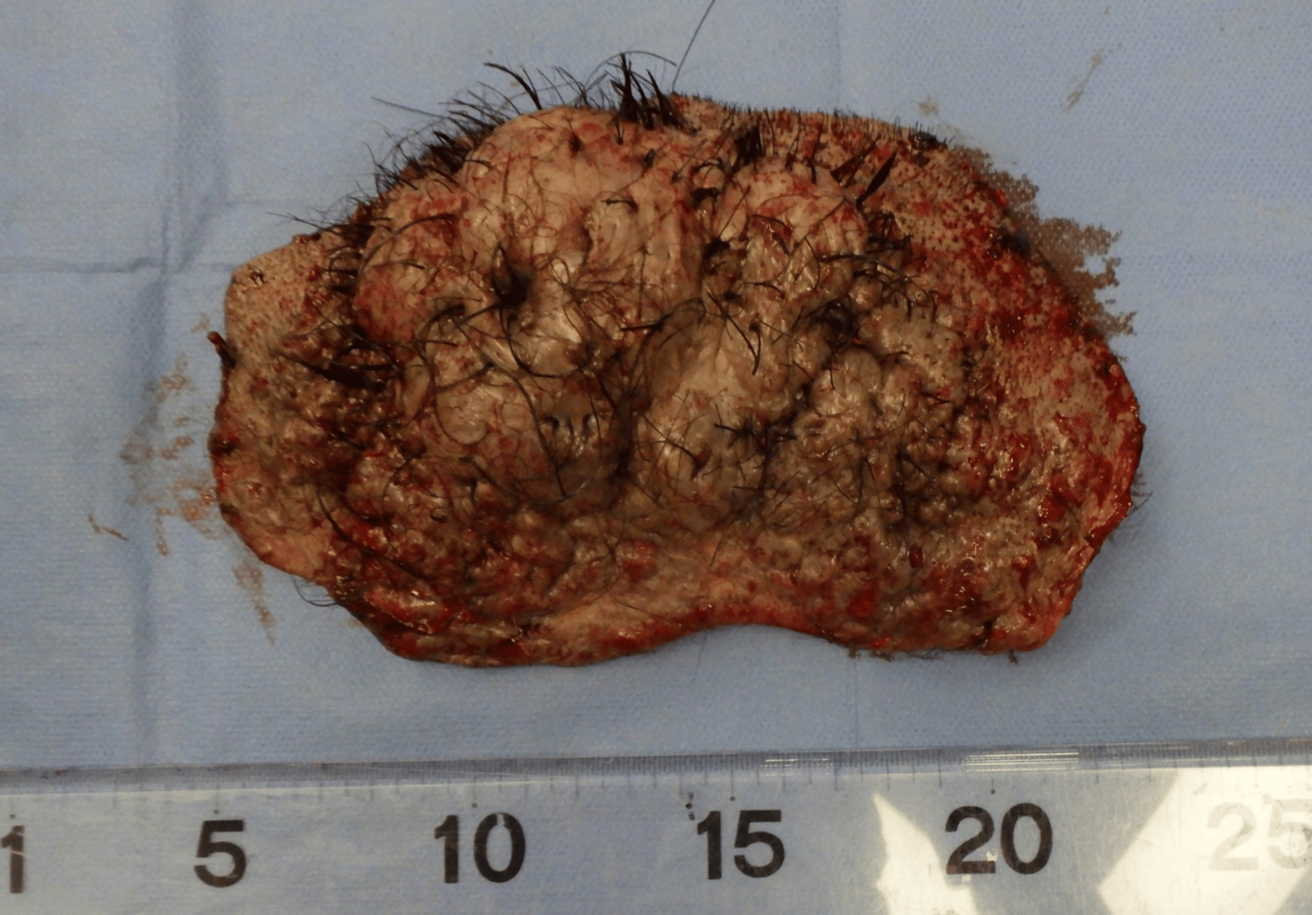
The resected tumor The tumor was resected at the middle layer of the subcutaneous fat

**Figure 5:**
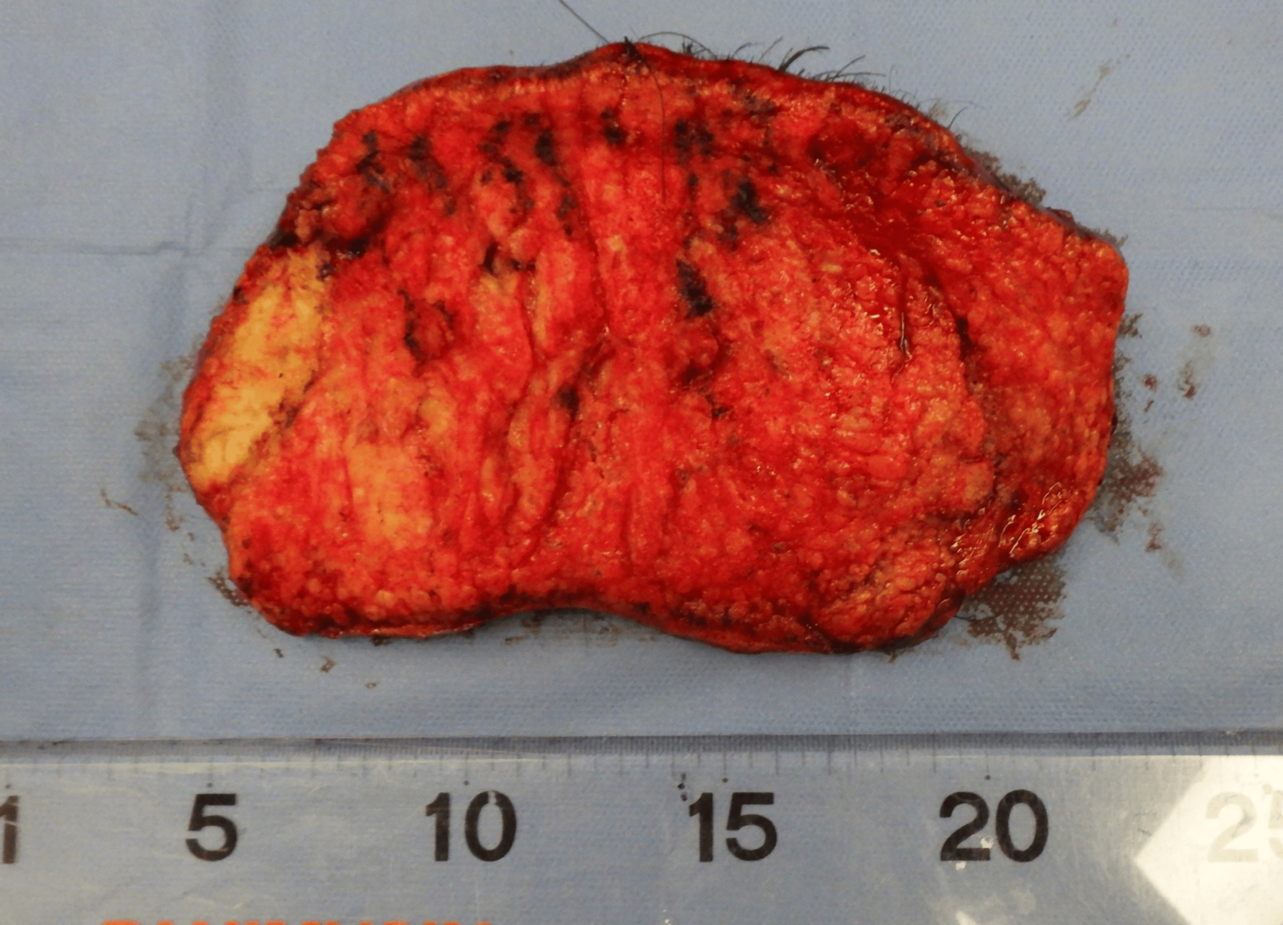
The resected tumor Photograph of the posterior side of the tumor

**Figure 6:**
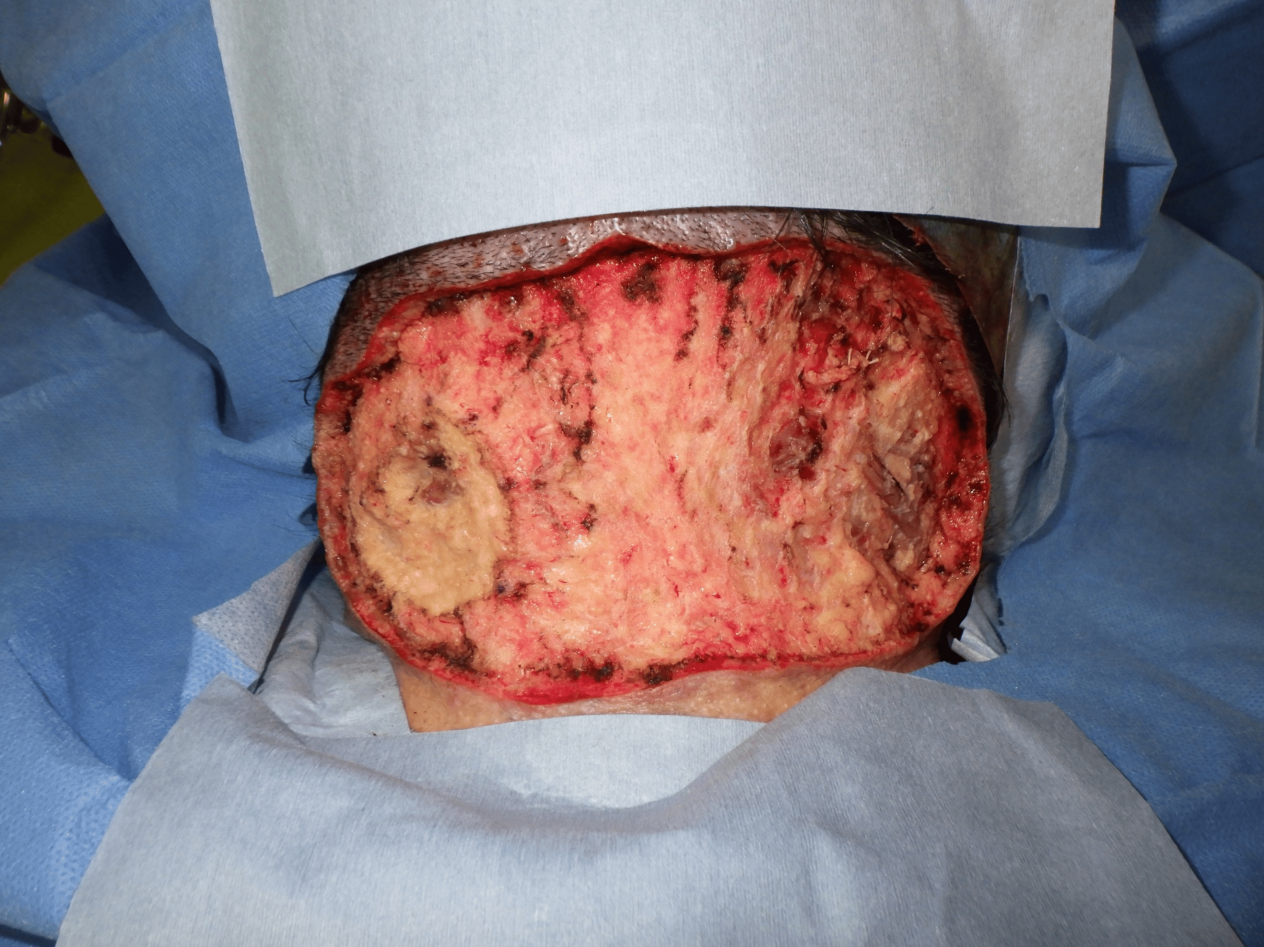
Operative findings The recipient site after the tumor resection

**Figure 7:**
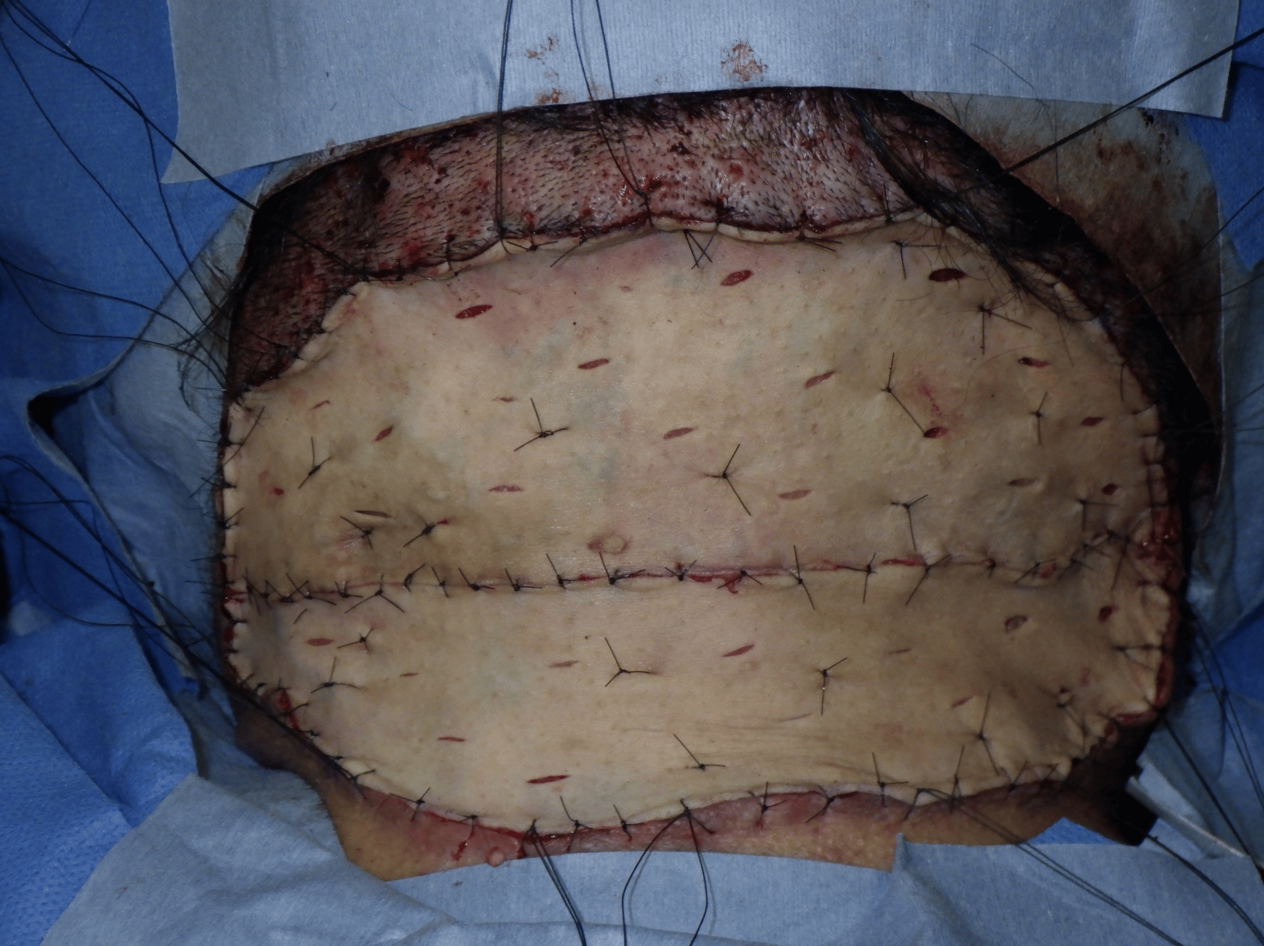
Postoperative findings Postoperative view of full-thickness skin grafts

Surgery was performed under general anesthesia. The entire lesion was excised, with deep margins extending to the mid-layer of the subcutaneous fat (Figures 4, 5), resulting in a 22 × 13 cm skin defect. Full-thickness skin grafts were subsequently harvested from both inguinal regions and transplanted to the defect area (Figures 6, 7).

**Figure 8:**
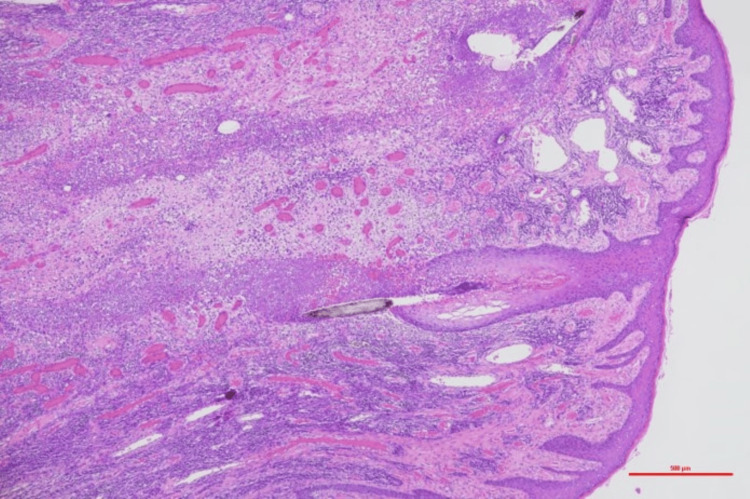
Pathological findings Hyperkeratosis, follicular destruction, and abscess formation were observed. Hematoxylin and eosin (HE) staining (scale bar = 500 μm)

**Figure 9:**
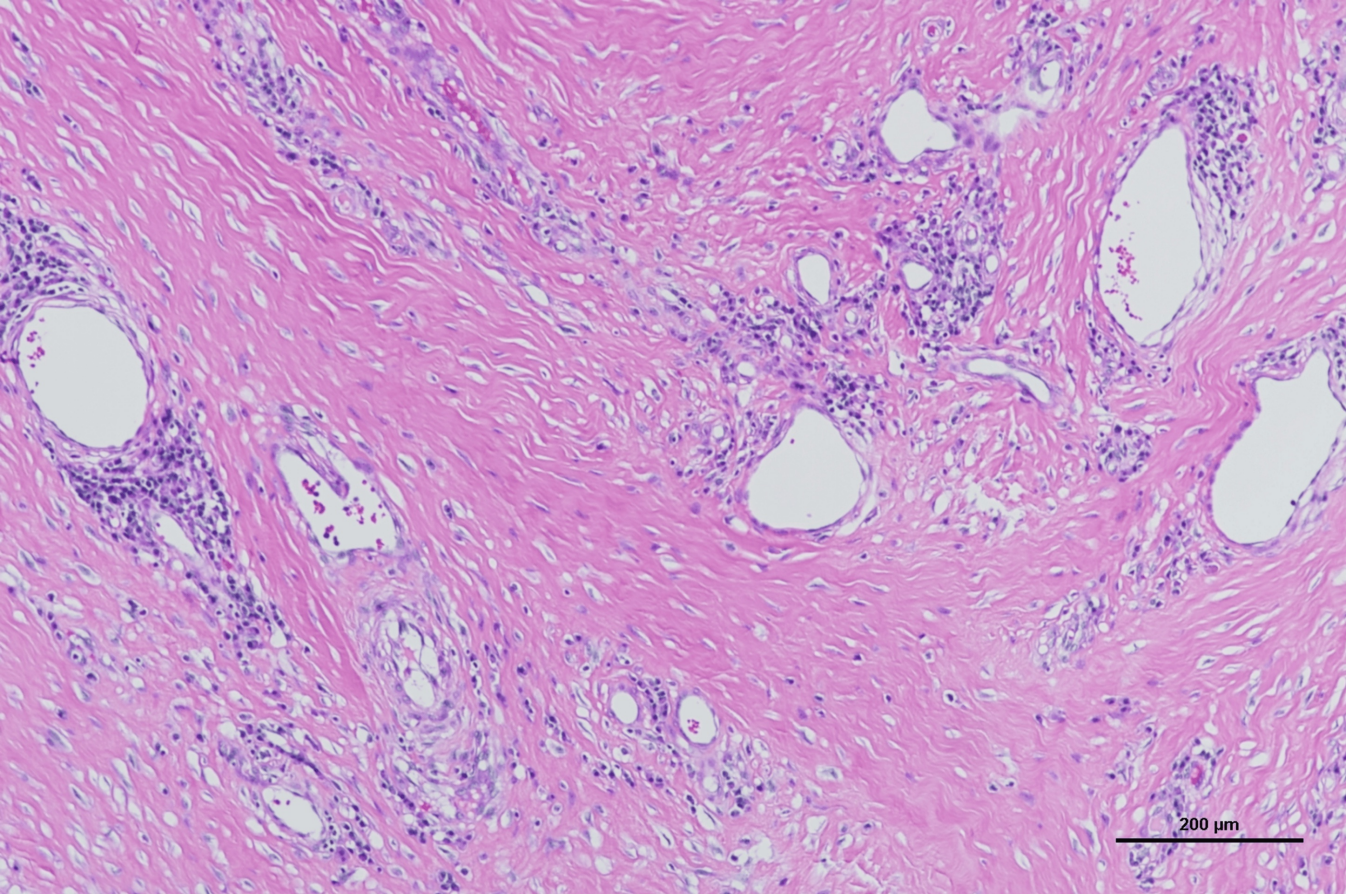
Pathological findings The dermis exhibited marked collagen fiber thickening and severe fibrosis. Hematoxylin and eosin (HE) staining (scale bar = 200 μm)

The histopathological examination of the excised specimen revealed hyperkeratosis, follicular destruction, and abscess formation. The dermis exhibited marked collagen fiber thickening and severe fibrosis, leading to the diagnosis of AKN (Figures 8, 9).

**Figure 10:**
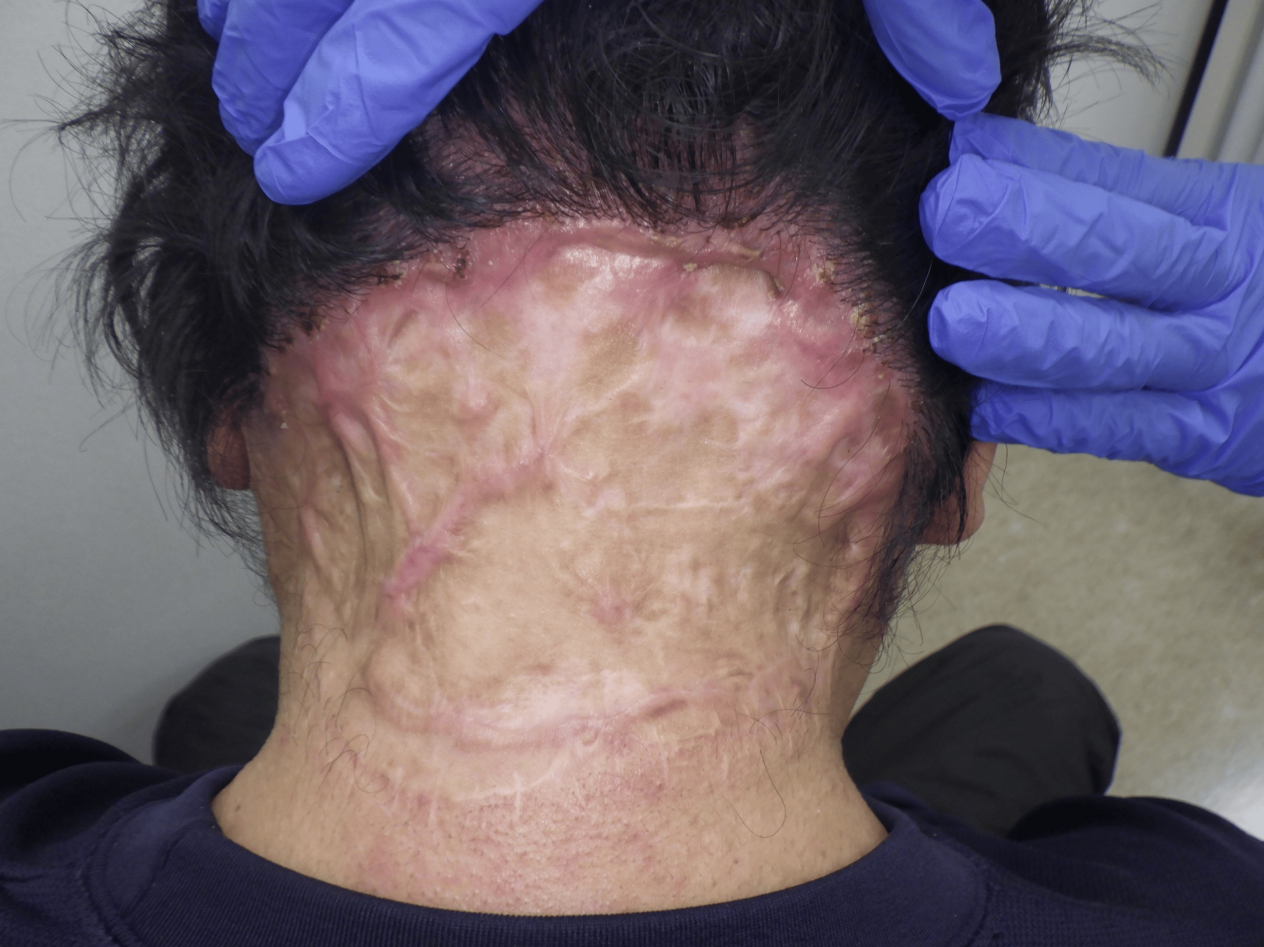
Clinical appearance after surgery Postoperative view after 10 months of follow-up. There was no recurrence on the occipital region of the patient’s scalp

At the patient’s 10-month postoperative follow-up, no recurrence was observed, and he demonstrated a favorable recovery (Figure 10).

Case 2

**Figure 11:**
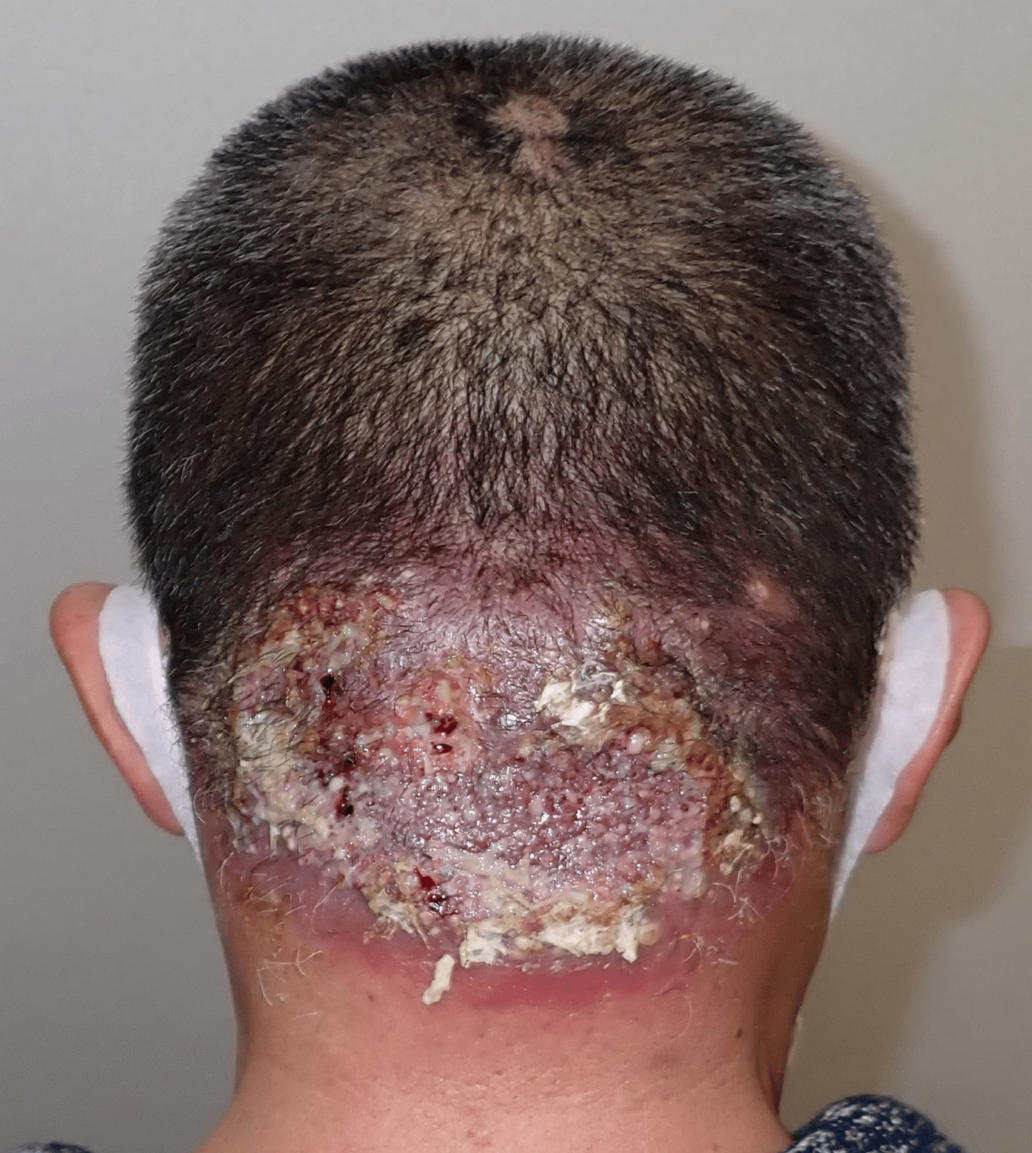
Postoperative photo An 8 × 6 cm lesion with swelling and purulent discharge was observed extending from the occipital region of the scalp to the posterior neck

A 61-year-old man presented with a mass in the occipital region of his scalp that he had been aware of for several years; however, his symptoms had worsened a month before his visit, accompanied by pus discharge, prompting him to be evaluated. His medical history included diabetes mellitus and hypertension, for which he was taking anagliptin and imidapril, respectively. A physical examination revealed a 10 × 12 cm lesion with swelling and purulent drainage extending from the occipital region of the scalp to the posterior neck (Figure 11).

**Figure 12:**
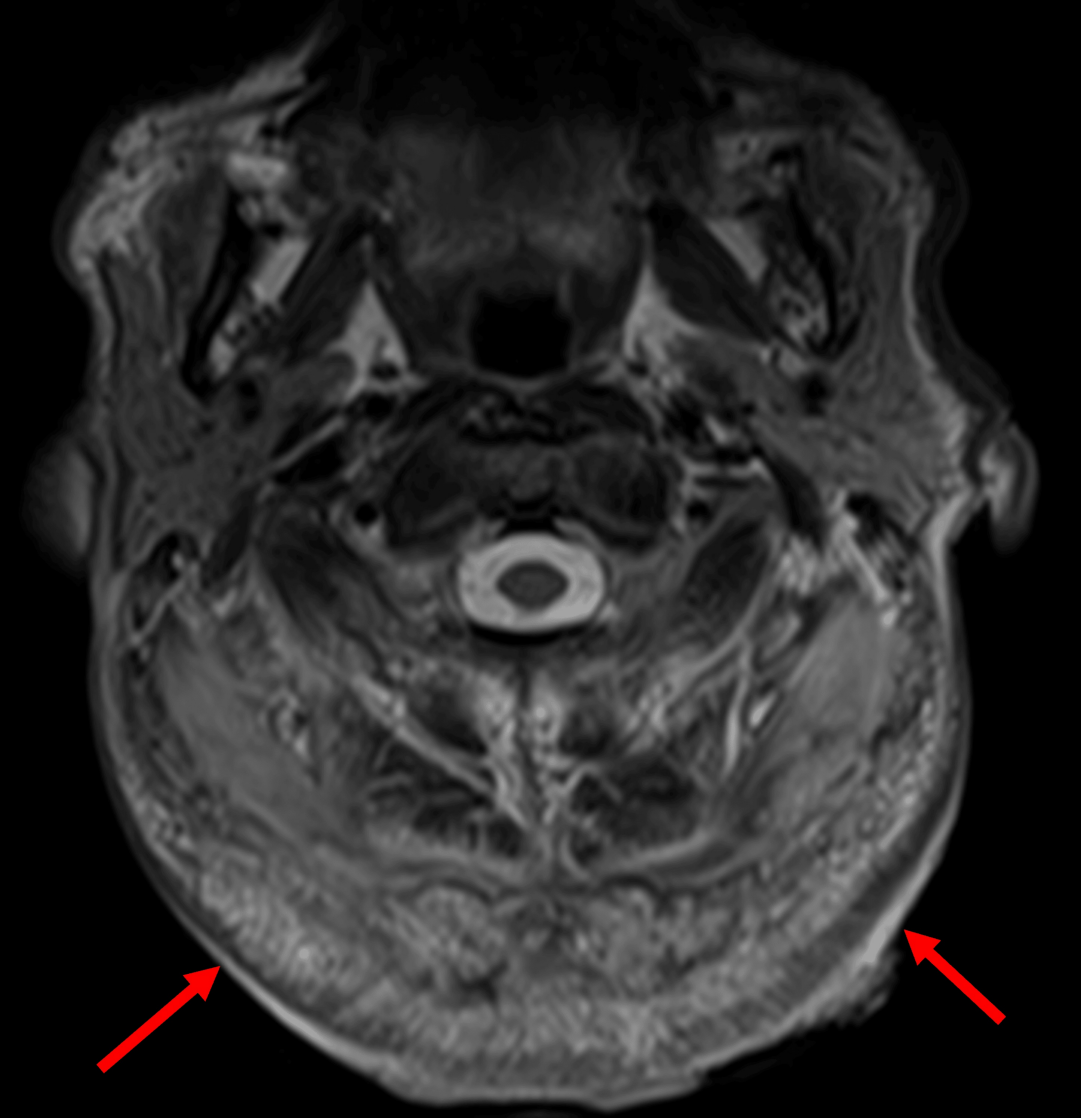
Preoperative MRI T2-weighted magnetic resonance imaging (MRI) revealed a high-signal area extending to the muscle layer Red arrow: tumor

**Figure 13:**
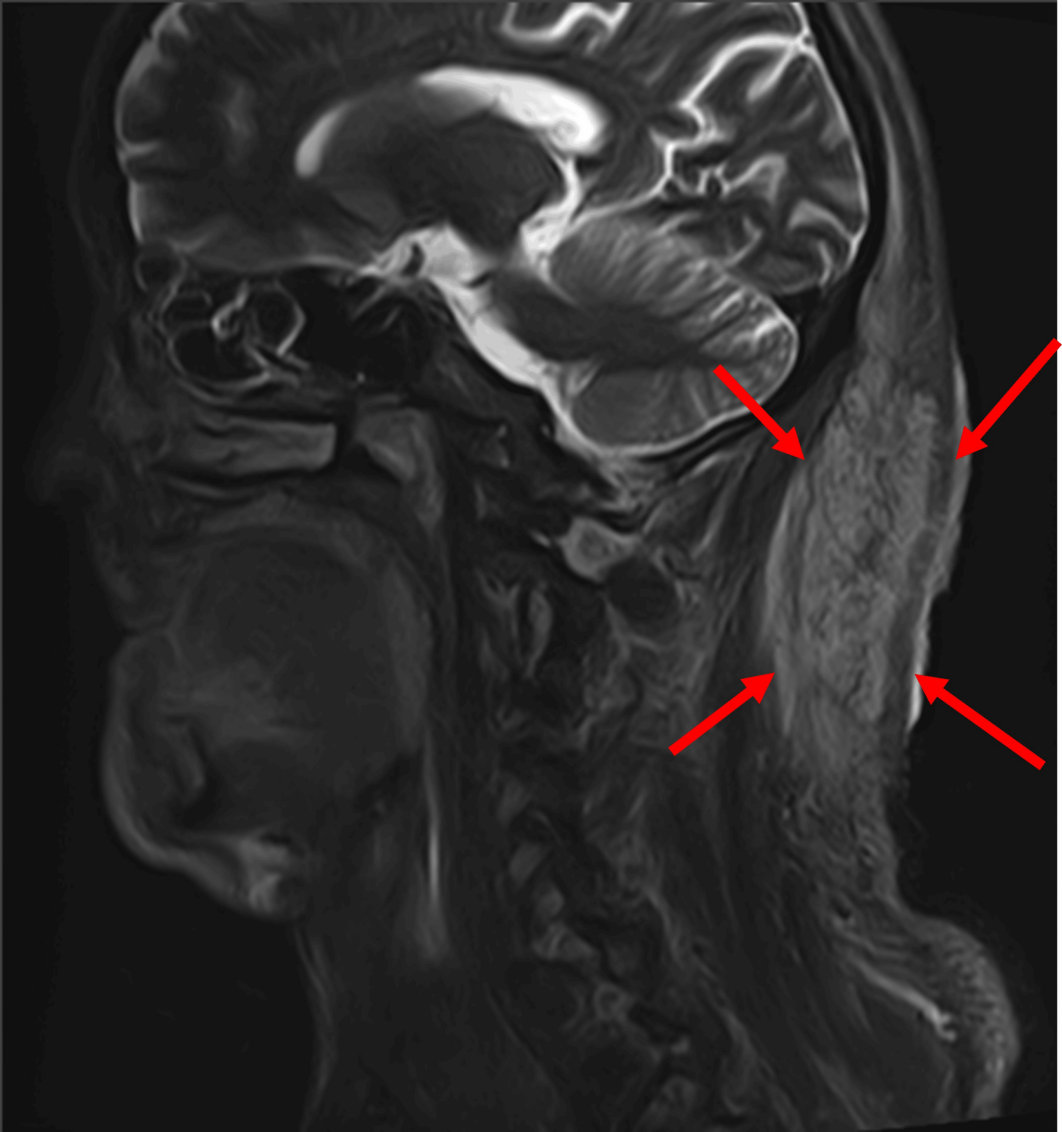
Preoperative magnetic resonance imaging (MRI) Sagittal view of the MRI

Laboratory findings showed a white blood cell count of 11,600/µL, random blood glucose of 260 mg/dL, hemoglobin A1c of 13.1%, and C-reactive protein of 2.83 mg/dL, indicating poor glycemic control and mild inflammation. T2-weighted MRI revealed a high-signal area infiltrating the muscle layer (Figures 12, 13).

At the initial visit, an incision was made at the lesion site, from which a tissue biopsy revealed abundant collagen fibers and marked neutrophil infiltration. Due to the extent of the lesion and poor granulation tissue with purulent discharge, surgical treatment was planned to remove the affected tissue.

**Figure 14:**
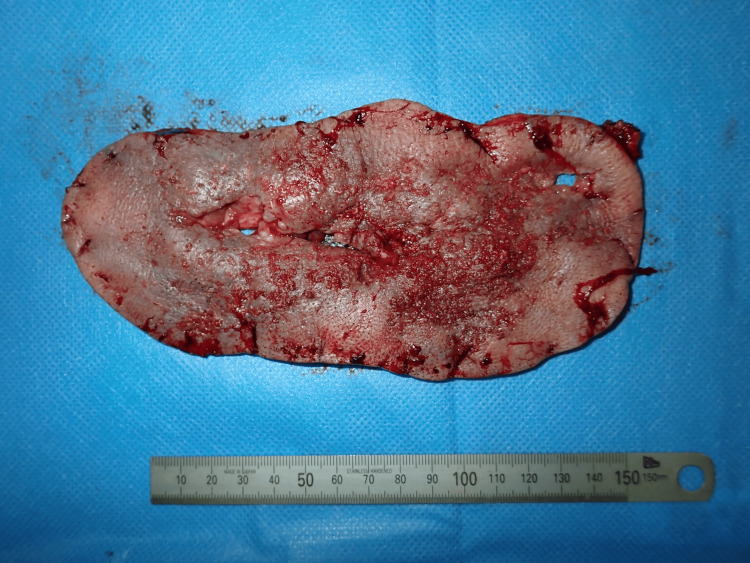
The resected tumor The tumor was resected above the fascia. In areas where inflammation had caused fascial disruption, resection was performed below the fascia

**Figure 15:**
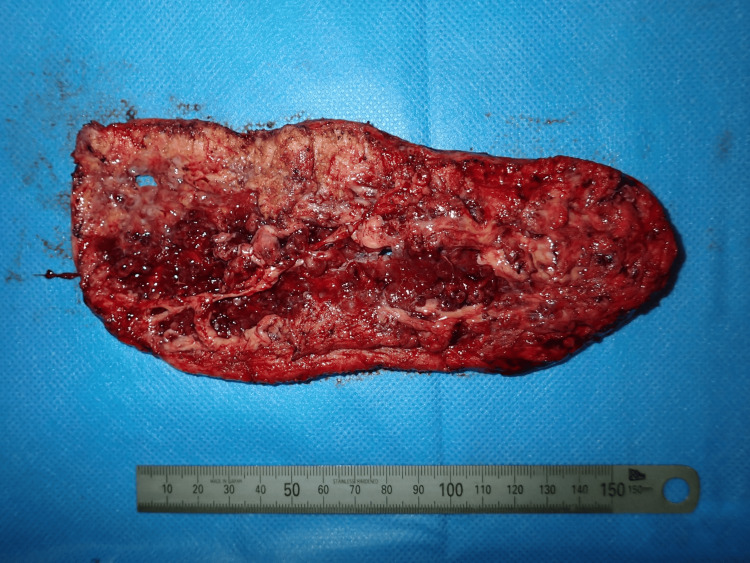
The resected tumor Photograph of the posterior side

The tumor was excised along its margins and the fascia; however, due to the presence of deep inflammation, parts of the fascia were dissolved, necessitating the removal of inflamed areas partially below the fascia (Figures 14, 15).

**Figure 16:**
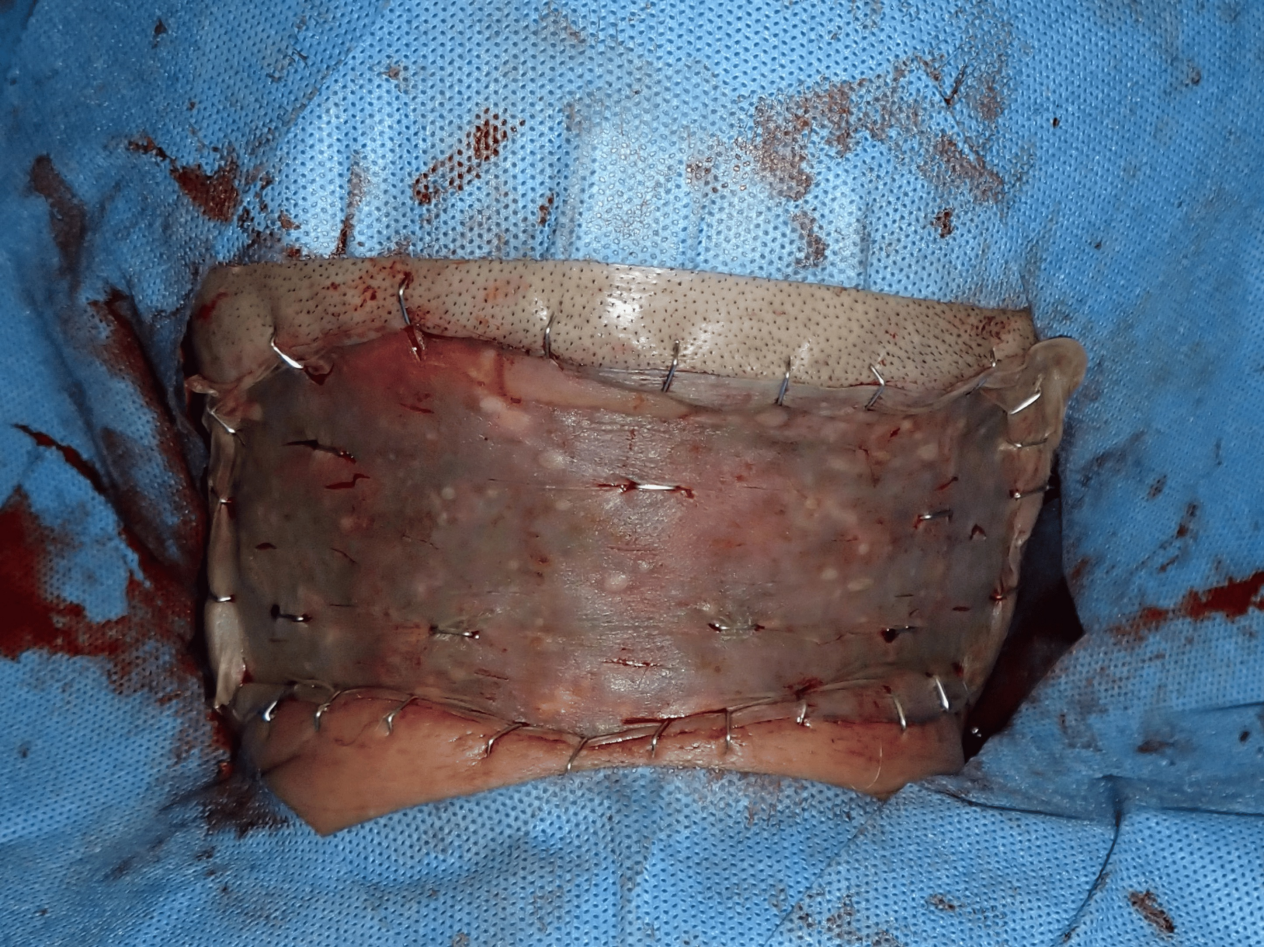
Postoperative findings A split-thickness skin graft was harvested from the posterior thigh

After the inflammation subsided, 26 days post-tumor excision, a secondary skin graft was performed. The skin defect measured approximately 6.5 × 15 cm; therefore, a split-thickness skin graft was harvested from the posterior thigh and transplanted at the defect site (Figure 16).

**Figure 17:**
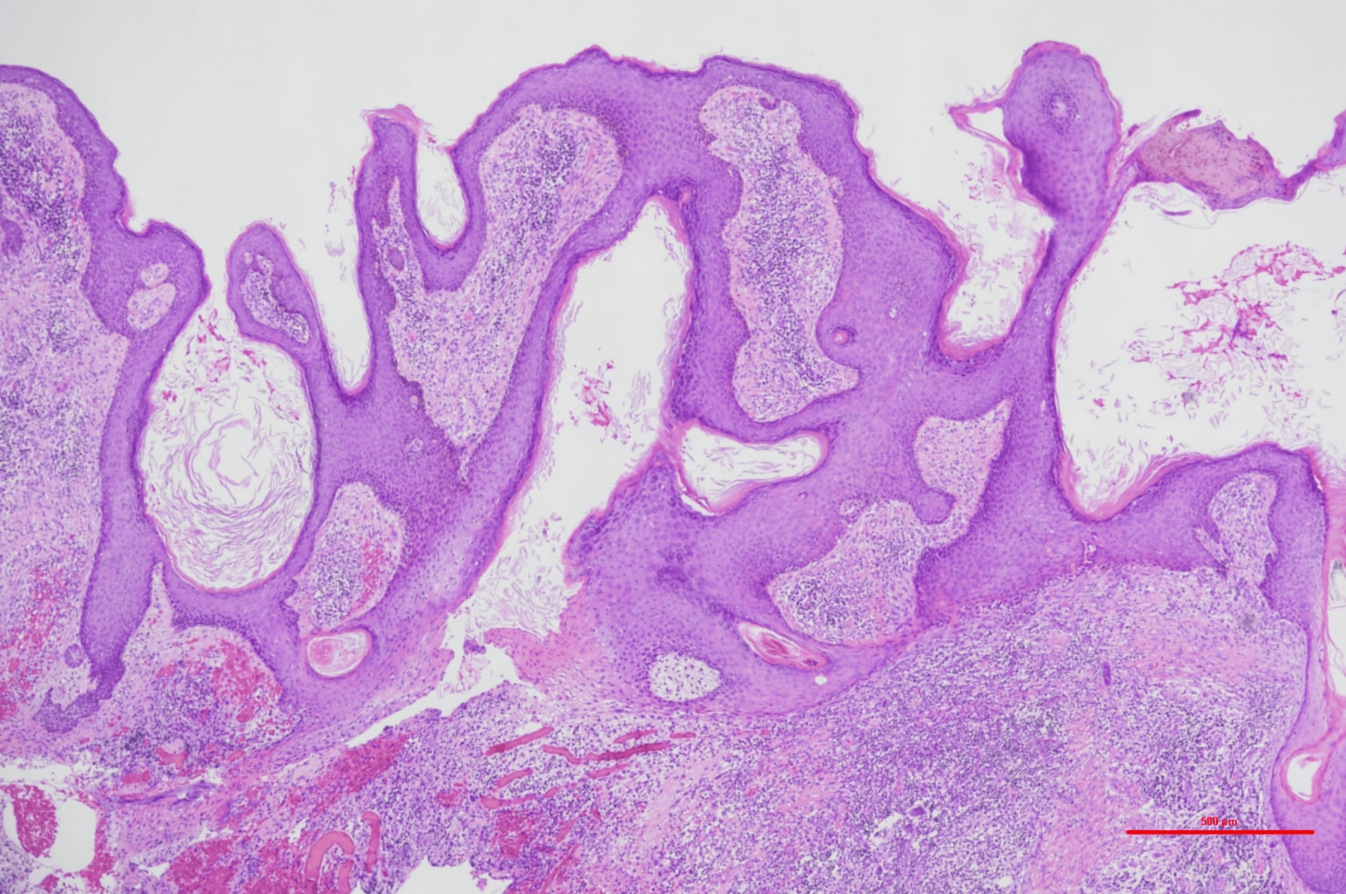
Pathological findings Papillomatous epidermal hyperplasia, irregular elongation of the rete ridges, and hyperkeratosis were observed. Hematoxylin and eosin (HE) staining (scale bar = 500 μm)

**Figure 18:**
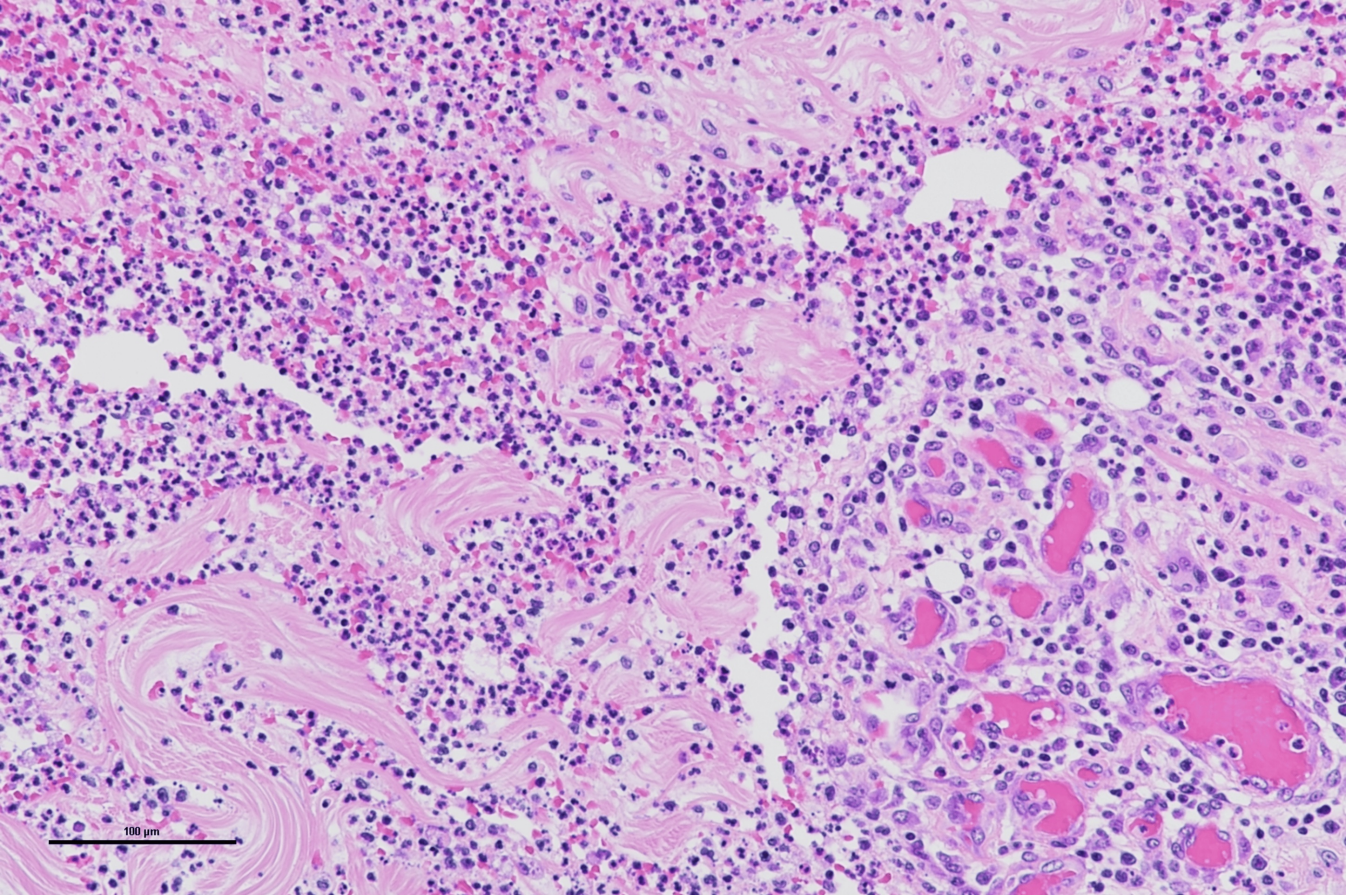
Pathological findings Dermal fibrosis and granulation tissue formation were present, accompanied by foreign body granulomas. Hematoxylin and eosin (HE) staining (scale bar = 100 μm)

The histopathological examination of the excised specimen showed papillary hyperplasia of the epidermis, irregular elongation of the rete ridges, hyperkeratosis, dermal fibrosis, and granulation tissue formation, accompanied by foreign body granulomas (Figures 17, 18).

**Figure 19:**
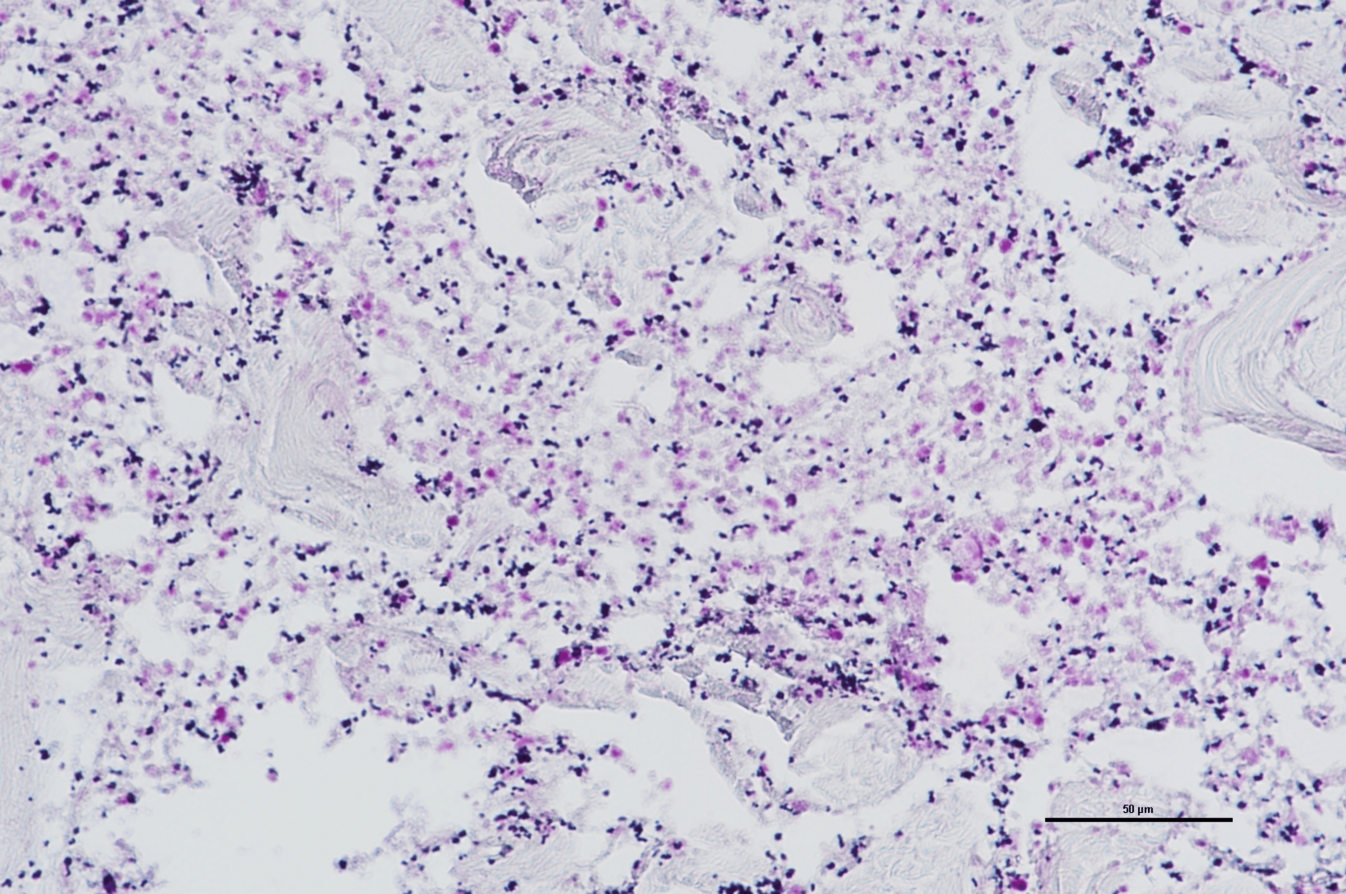
Pathological findings Degeneration of collagen fibers and neutrophilic infiltration were observed, along with the presence of Gram-positive cocci. Gram staining (scale bar = 50 μm)

Collagen fiber degeneration and neutrophil infiltration were observed, along with Gram-positive cocci, leading to a diagnosis of DCS (Figure 19).

**Figure 20:**
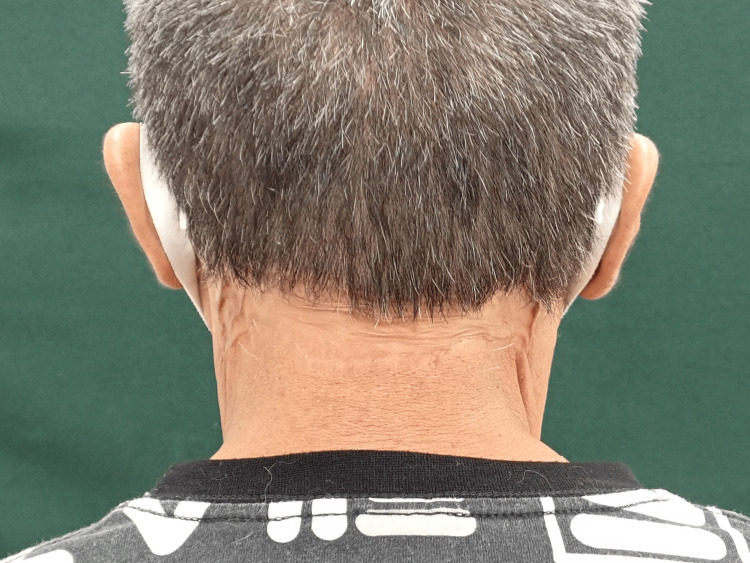
Clinical appearance after surgery Postoperative view after 5 months of follow-up. No recurrence was observed, and a favorable cosmetic outcome was achieved

At the patient’s five-month postoperative follow-up, no recurrence was observed, and the patient had achieved cosmetically acceptable outcomes (Figure 20). 

## Discussion

Severe chronic suppurative diseases of the scalp are classified into conditions such as DCS, AKN, and FD. These conditions share common features, including chronic scarring, folliculocentric pustules, and responsiveness to antibiotic treatment. Based on these shared characteristics, a broader concept, chronic folliculocentric pustulosis of the scalp, has been proposed to encompass these diseases [1].

AKN is reportedly more common among individuals of African descent, while it is relatively rare among Asian individuals. Similarly, DCS is also an uncommon form of chronic suppurative scalp disease [2,3]. These conditions involve the development of progressive inflammation associated with follicular obstruction and destruction, with some advanced cases being difficult to treat. In the cases presented herein, surgical treatment yielded favorable outcomes for the aforementioned conditions.

AKN is a chronic inflammatory disease primarily affecting the posterior neck and occipital area of the scalp. Characterized by pustules and papules associated with scarring, AKN eventually leads to scarring alopecia [4]. In Case 1, the mass was associated with keloid formation, and histopathological findings revealed significant fibrosis resembling a keloid, consistent with AKN. Although the etiology of AKN remains unclear, mechanical trauma or irritation is believed to play a role [5]. The patient in Case 1 routinely wore a helmet for work, which may have acted as a source of physical stimulation. Treatments for AKN include systemic and topical antibiotics, local steroid injections, and radiotherapy [6]; however, surgical excision is preferred for severe cases [7]. While complete recurrence after surgical excision has not been reported, some cases have exhibited mild postoperative recurrences, such as pustules and papules, which require treatment with topical steroid therapy [8].

DCS, also known as perifolliculitis capitis abscedens et suffodiens or Hoffmann disease, is a rare chronic inflammatory scalp condition characterized by recurrent folliculitis, painful and fluctuating scalp abscesses, fistula formation, and scarring. Epidemiologically, DCS is more common in male patients, with nodules and abscesses frequently appearing on the vertex or occipital region of the scalp. Approximately half of the patients affected by DCS develop lesions in the occipital region of the scalp [9]. In Case 2, the findings of recurrent folliculitis, abscesses, and fistula formation were consistent with DCS. Treatment for mild DCS cases includes topical therapies such as antibiotics, steroids, and benzoyl peroxide. In recent years, the efficacy of immunosuppressants and biologic agents, particularly tumor necrosis factor inhibitors (adalimumab, infliximab), has been demonstrated [10,11]. These agents, proven effective in the treatment of related conditions such as hidradenitis suppurativa, are also thought to be beneficial for DCS; however, in severe cases or those with extensive lesions, surgical intervention is recommended [12]. Uncontrolled DCS is a risk factor for a variety of complications such as squamous cell carcinoma and osteomyelitis of the skull due to chronic inflammation, highlighting the need for early treatment [13].

Although AKN and DCS can be differentiated based on histopathological findings, some cases of AKN may coexist with DCS [14], suggesting that these conditions cannot be entirely separated as distinct entities. Both conditions commonly affect the occipital region of the scalp. For severe cases, surgical excision and skin grafting are necessary; however, this treatment may result in hair loss at the resection site. With appropriate treatment, the resection site is relatively inconspicuous when hidden by surrounding hair. By appropriately selecting cases, cosmetically superior reconstruction outcomes can be achieved.

## Conclusions

We encountered two cases of chronic suppurative diseases of the scalp treated with tumor excision and skin grafting, both of which achieved cosmetically favorable outcomes. Although rare, attempting medical treatment in the early stages of these diseases and performing surgical intervention for cases resistant to medical therapy can significantly improve patients’ quality of life. To establish the appropriate criteria for early surgical intervention and evaluate possible outcomes, further accumulation and analysis of cases are necessary.
